# Spring Medicine

**Published:** 1875-03

**Authors:** 


### SPRING MEDICINE.

An excellent friend wishes us to tell our readers of "some good, simple medicine, to purify the blood in Spring." Now, we like our friend, and we are bound to treat whatever request or suggestion he may make with courtesy and respect. But he must not deem us severe if we tell him that there is a good deal of folly in the old idea of a regular Spring physicing, among people who live anywhere near right.

People complain of lassitude and weakness and lack of energy and strength in the Spring, and suppose they ought to take something. They remember the "picra," or senna and salts, or sulphur and molasses of childhood, and incline to practice on the ancient plan. They are certain they do not feel as vigorous at the first return of mild days as they did when the air was sharp and cold. They become more quickly exhausted, and want to sit down oftener to rest.

These sensations are quite general, we are aware; but they need not be. If we have lived wisely and well through the cold term, and if we are doing our duty by ourselves now, we can not honestly declare that we need medicine. If we have lived largely out of doors for months; if we have warmed ourselves as often by exercise, by motion, by active exertion, as by artificial heat; if we have slept only in a room with a window opened to admit the pure outer air; if we have depended more upon wholesome grains, vegetables, and fruits, than we have upon pork and highly-seasoned foods; if we have washed ourselves all over daily; if we have left exposed to pure air through the night all the garments worn through the day; if we have avoided alcohol and tobacco as the destructive and abominable poisons which they are,—we are not in need of doctors nor drugs, and we know it.

If we have been weak, or lazy, or careless, or wicked, in these directions; if we have left undone the many trifling but important things which we ought to have done to secure for ourselves health and comfort, and have done the foolish things which we ought not to have done,—then we may take physic, and imagine we are paying off the long score by a day or two of misery; while, in fact, we have, by false living, considerably abbreviated our term of life.

The man or woman or child who lives right, takes the very best purifying medicine every day and hour. Moreover, the medicine thus taken is the cheapest known. One kind—the most important, in fact—is absolutely free to all. It is put up in the very best and most convenient form for use, and is given away. Some unfortunate people live in neighborhoods where the supply is very small, and are unable to obtain as much as they ought to take of this valuable medicine; others are indifferent about it, and so get along for a time without it; while a very considerable number take it constantly, and decline to pass a single hour without an ample dose of it. The name of this very effective article is PURE AIR.

There is one fact connected with the lassitude felt in early Spring which all will do well to remember. We are compelled to burden ourselves with a good deal of clothing in the Winter, and when the warmer days of

Spring come we are still carrying from twenty to forty pounds of clothing. If we would lessen the weight of our clothing promptly, as the season advances, we should find that the weariness which we had experienced simply came from the burden of our dress. If we would exchange the heavy overcoat for a light one when we walk out in the sunshine of a mild day, and return to the heavy garment on the advent of a cold snap, we should find that the vigcr and energy of Winter had not passed from us.

But we can not keep our cake after we have eaten it. We can not go to bed at midnight and rise up fresh and strong at sunrise. We can not smoke our poisonous weeds and drugs, and have a clear, strong, powerful brain, for several hours afterwards. We can not drink whiskeys and ales and other indigestible things and keep the digestive instruments in perfect tune. We can not go unwashed and be sweet and clean and pure. We can not sleep in close rooms all winter and be well and strong and energetic when Spring comes. We can not live largely on oily substances—unless our life is active and remote from the influence of artificial heat—and enjoy strong stomachs and abundant appetites, as the cold weather departs.

If we have lived unwisely through the Winter, let us not fly to drugs. Let us simply reform. Let us try a diet of fruit and grain and vegetables, for a month. Let us see if we can not live without fat things. Let us try the experiment of living on half our usual quantity of food. Let us throw open our windows by day, and, above all things, by night. Let us shift our quarters from a north, sunless outlook, to a warm, sunny, southern exposure. Let us tear down the curtains and unhinge the shutters. Let us live for a while like sensible human beings, and see if we are not well enough without other "Spring Medicines."

---

Teachers succeed best if they are good tempered and appreciative. The average child is susceptible, and responds promptly to generosity, confidence, and obvious good will. When the child-heart feels that the teacher wishes, expects, and enjoys goodness in the pupil, it has the strongest motive to be good. But let the dark suspicion once get into the mind, "My teacher likes me to fail and trip, for the satisfaction of being down upon me," and that little is thenceforth learned is the less of two evils. The greater and graver is the embittering of the heart, the poisoning of its fountains, and the kindling of a malignant, vindictive spirit, within it, of which the effect is to obliterate all that might be angelic, and to produce all that is demonaical.

---

There is a Spanish proverb that a tradesman who would grow rich must buy of those who go to be executed, as not caring how cheap they sell; and sell to those who go to be married, as not caring how dear they buy.

---

A man would have no pleasure in discovering all the beauties of the universe, even in heaven itself, unless he had a partner to whom he might communicate his joys.—*Cicero.*

---

Slow and sure is better than fast and flimsy.

